# Zinc as a protective factor against COPD: Insights from observational and Mendelian randomization analyses

**DOI:** 10.1097/MD.0000000000043340

**Published:** 2025-08-08

**Authors:** Banghui Liu, Jinhe Yuan, Lu Huan, Li Peng

**Affiliations:** a Outpatient Department, Renji Hospital, School of Medicine, Chongqing University, Chongqing, China; b Department of Respiratory and Critical Care Medicine, Renji Hospital, School of Medicine, Chongqing University, Chongqing, China; c Department of Hepatobiliary and Pancreatic Surgery, Chongqing Fifth People’s Hospital, Chongqing, Chongqing, China.

**Keywords:** COPD, Mendelian randomization, micronutrients, restricted cubic spline, zinc

## Abstract

Chronic obstructive pulmonary disease (COPD) remains a major global health burden, with limited effective preventative strategies. Zinc, a key micronutrient, plays critical roles in immune regulation, anti-inflammatory processes, and maintaining epithelial integrity, yet its relationship with COPD and related infections remains poorly understood. This study utilized data from the National Health and Nutrition Examination Survey and Mendelian randomization (MR) analyses. Serum zinc levels were assessed in 67,502 participants, with COPD diagnosed based on forced expiratory volume in 1 second/forced vital capacity <0.70 or physician reports. Weighted logistic regression and restricted cubic spline analyses explored nonlinear associations between zinc exposure and COPD risk. Stratified analyses examined the role of age, race, and smoking status as modifiers. MR analyses were conducted to assess causality, with sensitivity tests ensuring robustness. Higher zinc levels were associated with reduced COPD risk, showing significant nonlinear dose–response patterns (*P*-overall < .001; *P*-nonlinear = .030). Stratified analyses revealed stronger protective effects in individuals aged ≤ 65 years (odds ratio [OR] = 0.963, *P* = .003), Non-Hispanic Whites (OR = 0.965, *P* = .051), and smokers (OR = 0.965, *P* = .023). MR analysis confirmed Zinc’s causal protective role in reducing COPD-related infections (OR = 0.979, *P* = .042), independent of confounders. This study provides robust evidence supporting zinc as a protective factor against COPD and related infections, emphasizing its potential as a therapeutic target for prevention and management. Future studies should explore optimal zinc supplementation strategies and gene–environment interactions to maximize its clinical benefits.

## 1. Introduction

Chronic respiratory diseases (CRDs) are one of the 4 major chronic diseases affecting human health identified by the World Health Organization.^[1]^ They represent a group of disorders impacting the respiratory tract and related structures. The most common types include chronic obstructive pulmonary disease (COPD), asthma, and interstitial lung disease. Chronic obstructive pulmonary disease poses a significant public health challenge, with an estimated 544.9 million people affected globally as of 2017. It is projected that factors such as antimicrobial resistance, an aging population, and emerging pathogens will further escalate the disease burden.^[2,3]^ Consequently, identifying modifiable risk factors associated with these diseases related infection is essential.^[4]^ Numerous micronutrients have been shown to play critical roles in the immune system, being essential for immune cell proliferation and maturation, cytokine release,^[5]^ and for the function of antioxidant defense enzymes within immune cells.^[5]^

Zinc plays at least 4 critical roles in respiratory epithelial cells, acting as an antioxidant^[6]^; an anti-inflammatory agent^[7]^; an antiapoptotic factor^[8]^; and a key cofactor for DNA synthesis. Despite these benefits, zinc deficiency is recognized as a risk factor for pneumonia.^[9]^ Interestingly, Mendelian randomization (MR) analyses have shown no significant association between genetically predicted circulating zinc levels and pneumonia risk.^[10]^ A systematic review of randomized controlled trials found only limited evidence supporting the effect of micronutrient supplementation on respiratory infection risk, emphasizing the need for further research.^[11,12]^ Among these findings, one review specifically reported no effect of zinc supplementation on the incidence of respiratory tract infections in infants.^[11]^

MR is often portrayed as a “natural experiment,” leveraging inherent genetic variation to simulate exposure to specific conditions. This approach allows researchers to assess whether such exposures causally influence certain traits.^[13]^ By using single nucleotide polymorphisms (SNPs) as genetic proxies, MR provides a robust framework to establish causal relationships between exposures and outcomes. A major advantage of MR is that genetic variants are randomly allocated, which reduces potential biases from reverse causation and confounding (limitations often present in traditional observational studies).^[14,15]^ MR relies on 3 core assumptions: (1) genetic variants are associated with the exposure of interest; (2) these variants are not linked with confounding factors; and (3) the genetic variants influence the outcome solely through their effect on the exposure, without other mediating pathways. Given its strengths in minimizing bias and confounding, MR has become widely used in scientific research and clinical trial design.^[16]^ Despite previous studies on zinc and CRDs-related infections yielding inconsistent findings, often due to limited sample sizes and potential confounders, this study employs MR analysis to investigate the relationship more rigorously. By reducing confounding effects and mitigating reverse causation, MR analysis holds significant potential to identify causal links between diseases and traits.

Zinc, a key micronutrient involved in immune regulation, anti-inflammatory processes, and epithelial barrier maintenance, may offer potential benefits in reducing risk of COPD and related infections. However, its role in COPD and related infections has been inadequately explored. This study aimed to investigate the association between zinc exposure and risk of COPD and related infections, using data from National Health and Nutrition Examination Survey (NHANES) and MR to strengthen causal inference. By addressing confounding factors and examining nonlinear dose–response relationships, this research highlights the potential of zinc as a modifiable factor in COPD and related infections prevention, providing a basis for future dietary guidelines and therapeutic interventions.

## 2. Materials and methods

### 2.1. Study population

This cross-sectional study used data from the NHANES conducted from 2021 to 2023. NHANES is a publicly available database that collects health-related data through interviews, physical examinations, and laboratory tests. Ethical approval was obtained from the National Center for Health Statistics’ Ethics Review Board, and all participants provided written informed consent. A total of 94,086 participants were initially included. Subjects were excluded if they: (1) lacked serum zinc measurements (n = 6308); (2) participants missing data on gender\age\race\education (n = 20,149); (3) had missing marriage\income data (n = 35); or (4) had incomplete smoking status (n = 92). After exclusions, 67,502participants remained for analysis (Table 1).

**Table 1:** The baseline characteristics by quartiles of the COPD: National Health and Nutrition Examination Survey 2021–2023.

		Overall(%)	Without COPD (%)	With COPD (%)	*P*-value
N		67,502	57,097	10,405	
Gender (%)	Female	38,232 (56.6)	29,853 (52.3)	8379 (80.5)	<.001
	Male	29,270 (43.4)	27,244 (47.7)	2026 (19.5)	
Age (mean [SD])		57.04 (16.32)	55.74 (16.59)	64.21 (12.52)	<.001
Race (%)	Mexican American	4075 (6.0)	3756 (6.6)	319 (3.1)	<.001
	Non-Hispanic Black	6618 (9.8)	5875 (10.3)	743 (7.1)	
	Non-Hispanic White	43,880 (65.0)	36,300 (63.6)	7580 (72.8)	
	Other Hispanic	5944 (8.8)	5315 (9.3)	629 (6.0)	
	Other race – including multiracial	6985 (10.3)	5851 (10.2)	1134 (10.9)	
Education (%)	9–11th grade	3852 (5.7)	3296 (5.8)	556 (5.3)	<.001
	College graduate or above	29,511 (43.7)	25,104 (44.0)	4407 (42.4)	
	High school graduate	12,054 (17.9)	10,123 (17.7)	1931 (18.6)	
	<9th grade	2180 (3.2)	1933 (3.4)	247 (2.4)	
	Some college or AA degree	19,905 (29.5)	16,641 (29.1)	3264 (31.4)	
Marry (%)	Married/living with partner	38,657 (57.3)	32,857 (57.5)	5800 (55.7)	<.001
	Never married	11,697 (17.3)	10,425 (18.3)	1272 (12.2)	
	Widowed/divorced/separated	17,148 (25.4)	13,815 (24.2)	3333 (32.0)	
Income (mean [SD])		3.20 (1.62)	3.20 (1.63)	3.21 (1.59)	.507
Exposure zinc (mean [SD])		0.61 (1.39)	0.62 (1.40)	0.56 (1.34)	<.001
Smoke (%)	No	40,540 (60.1)	34,484 (60.4)	6056 (58.2)	<.001
	Yes	26,962 (39.9)	22,613 (39.6)	4349 (41.8)	

This table presents the baseline characteristics of the study population (N = 67,502), stratified by COPD status (with COPD, N = 10,405; without COPD, N = 57,097). Gender, age, race, education level, marital status, income, zinc exposure, and smoking status were analyzed. Continuous variables are presented as mean (standard deviation), while categorical variables are presented as frequency (%). *P*-values were derived from chi-squared tests for categorical variables and t-tests for continuous variables.

COPD = chronic obstructive pulmonary disease.

### 2.2. Exposure, outcome, and covariates

The primary exposure was serum zinc concentration (μg/dL), measured through standardized NHANES protocols. Zinc levels were divided into quartiles (0% = 0 μg/dL, 25% = 0.03 μg/dL, 50% = 0.14 μg/dL, 75% = 0.62 μg/dL) based on population distribution to explore dose–response relationships. Considering age specificity for COPD, study was divided into subgroups (age ≤ 65, age > 65) based on population distribution to explore dose–response relationships.^[4]^ Restricted cubic spline (RCS) regression was used to evaluate potential nonlinear associations between zinc levels and COPD risk. In addition, the primary outcome was COPD diagnosis, determined based on forced expiratory volume in 1 second/forced vital capacity ratio < 0.70 or self-reported physician diagnosis. What’s more, the analysis of covariates adjusted for potential confounders, including: gender, age, race, education, marriage, and smoke.

### 2.3. Statistical analysis

Baseline characteristics were summarized using weighted means (standard errors) for continuous variables and weighted percentages for categorical variables. Group differences were tested using ANOVA (continuous variables) and Rao-Scott *χ*² tests (categorical variables). Weighted logistic regression models were used to investigate the associations between Zinc and COPD. RCS regression was applied to assess nonlinear associations between zinc levels and COPD risk. Three models were constructed: Model 1. exposure + Gender + Age + Race + Education. Model 2. exposure + Gender + Age + Race + Education + Marry. Model 3. exposure + Gender + Age + Race + Education + Marry + income + Smoke. All analyses were conducted using R software. Statistical significance was defined as a two-sided *P*-value < .05. Visualization of RCS models was performed using the “rms” and “ggplot2” packages.

### 2.4. Data sources for MR analysis

**Figure 1.**
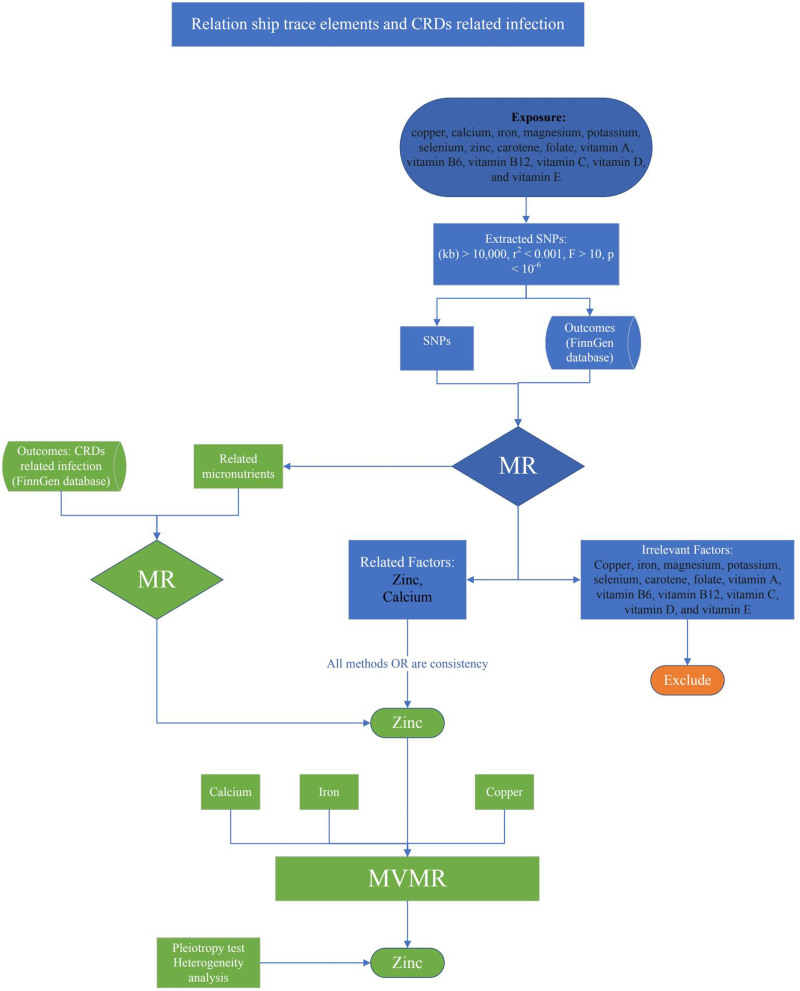
Relationship between trace elements and CRD-related infections. This diagram illustrates the associations between specific trace elements and CRD-related infections. The analysis evaluated various exposures, including copper, calcium, iron, magnesium, potassium, selenium, zinc, carotene, folate, and vitamins (A, B6, B12, C, D, and E), along with specific SNPs with stringent extraction criteria (kb > 10,000, *r*² < 0.001, *F* > 10, *P* < 10^‐6^). Using MR and MVMR methods, relevant factors like zinc and calcium were identified as significantly associated with CRD-related infections. Copper, iron, magnesium, potassium, selenium, carotene, folate, and vitamins A, B6, B12, C, D, and E were excluded as irrelevant. Consistency in odds ratios across methods was confirmed, with additional pleiotropy and heterogeneity analyses supporting the findings. The outcomes were derived from the FinnGen database. CRD = chronic respiratory disease, MR = Mendelian randomization, MVMR = multivariable MR, SNPs = single nucleotide polymorphisms.

The flowchart of the study was presented in Fig. 1. In summarize, a two-sample MR analysis was conducted using 15 exposure datasets derived from publicly available summary statistics in Open genome-wide association study (GWAS), and one outcome dataset from publicly available summary statistics in FinnGen. To reduce population stratification bias, exposure and outcome data were sourced from participants of European ancestry. The MR analysis adhered to 3 core assumptions. First, the instrumental variables (IVs) were associated with exposure. Second, the IVs were not associated with any confounders related to both the exposure and outcome. Third, the IVs affected the outcome only through their influence on the exposure.^[17,18]^

#### 2.4.1. Genetic epidemiologic data on micronutrients

In this study, we scoured the GWAS Catalog (https://www.ebi.ac.uk/gwas) and PubMed (https://www.ncbi.nlm.nih.gov/pubmed) for GWAS focused on blood-based concentrations of various minerals and vitamins. Our search yielded GWAS datasets on a comprehensive set of 15 micronutrients, including copper (ieu-a-1073), calcium (ukb-b-8951), carotenoids (ukb-b-16202), folate (ukb-b-11349), iron (ukb-b-20447), magnesium (ukb-b-7372), potassium (ukb-b-17881), selenium (ieu-a-1077), vitamin A (ukb-b-9596), vitamin B12 (ukb-b-19524), vitamin B6 (ukb-b-7864), vitamin C (ukb-b-19390), vitamin D (ukb-b-18593), vitamin E (ukb-b-6888), and zinc (ieu-a-1079). These datasets were employed as exposure factors within a two-sample MR analysis. To mitigate the impact of population stratification bias, all the selected studies were conducted using participants of European descent.

#### 2.4.2. Genetic epidemiologic data on essential hypertension

To gain genetic epidemiological insights into COPD and associated with infections, researchers utilized the FinnGen study database (FINNGEN, https://r11.finngen.fi/). A rigorous selection process considered factors such as sample size, sequencing depth, ethnic background, and the timing of data updates, resulting in the selection of a genomic dataset specific to COPD associated infections (https://storage.googleapis.com/finngen-public-data-r11). This dataset included 144,808 cases and 308,925 controls. Notably, all studies within the FINNGEN database had previously received ethical approval from relevant institutional review boards, with participants providing informed consent prior to data collection. Since this study only utilized publicly available, aggregate-level data, no additional ethical review was required.

### 2.5. Mendelian randomization analysis

This Mendelian randomization analysis tests the hypothesis that lifelong zinc exposure causally reduces COPD risk. Our approach relies on 3 core assumptions: 1. Genetic instruments validity: selected SNPs are robustly associated with serum zinc levels (prioritized by genome-wide significance thresholds and F-statistic validation to exclude weak instruments). 2. No confounding: these SNPs influence COPD risk exclusively through zinc-related pathways, independent of environmental factors (e.g., smoking) or demographic traits (e.g., ethnicity), leveraging the random allocation of alleles during meiosis. 3. No horizontal pleiotropy: SNPs exert effects solely via zinc modulation, not through alternative biological pathways (tested via MR-Egger regression and sensitivity analyses). The hypothesis aligns with zinc’s established biological roles in immune regulation and epithelial barrier maintenance (mechanisms plausibly modifying COPD pathogenesis). Analytical robustness is ensured by consistency across complementary MR methods (inverse-variance weighted, weighted median) and pleiotropy-robust sensitivity frameworks.

#### 2.5.1. Removal of weak IVs

To satisfy the requirements of the IV methodology, SNPs must be strongly associated with the exposure factor, independent of one another, and unaffected by outcome bias due to any imbalance in the exposure–outcome relationship. In this study, we employed R software to filter the SNP dataset rigorously, applying the following criteria: (1) SNPs designated as IVs were required to have a strong association with the exposure, indicated by a *P*-value threshold of less than 1 × 10^‐6^. Given the limited number of SNPs reaching genome-wide significance (*P* < 5 × 10^‐8^), this more relaxed threshold was used to increase the SNP pool for MR analysis, thereby enhancing the statistical power of the IVs; (2) SNPs were excluded if their *R*² coefficient exceeded 0.001 compared to the most significant SNP within a 10,000 kb window, where *R*² measures the variance in exposure explained by the SNPs, calculated as *R*² = 2 × (1 − MAF)² × MAF × (β₁/SD)², with MAF representing the minor allele frequency, β₁ denoting the allelic effect size for the exposure, and SD indicating the standard deviation; (3) SNPs were included if their F-statistic exceeded 10, where *F* = (N − 2) × *R*²/(1 − *R*²), with N representing the sample size, ensuring that selected SNPs were both strongly associated with the exposure and independent. To maintain consistency in the effect alleles across IVs, the study harmonized the summary statistics and excluded any palindromic SNPs.^[19]^

#### 2.5.2. Two-sample Mendelian randomization analysis

The IV data, derived using R software, were combined with effect sizes, after which both exposure and outcome data were preprocessed to ensure a consistent dataset structure. A two-sample MR analysis was then conducted, with the inverse variance weighted (IVW) approach as the primary method, supplemented by weighted median, MR-Egger, simple regression, and weighted regression analyses.^[20]^ These methods assessed the causal association between the exposure and outcome variables, with odds ratios (ORs) used to quantify the strength of the association.

The IVW method was a traditional statistical approach in MR that assumes all SNPs used in the analysis were valid IVs. Each of the 5 methods (IVW, weighted median, MR-Egger, simple regression, and weighted regression) offers distinct characteristics and assumptions, contributing to a comprehensive assessment of causal estimates. Following these analyses, tests for heterogeneity and confounding were conducted to ensure result robustness. 1. The *P*-value of the IVW method should be <.05, indicating a significant association between the IVs and the exposure; 2. The OR values should be directionally consistent across all 5 methods, suggesting reliable prediction of the outcome by the IVs; 3. The *P*-value for confounding should exceed .05, indicating no evidence of confounding by IVs. Following this filtering, the MR analysis is repeated on the exposure data linked to the outcome data. To assess horizontal pleiotropy, MR-Egger intercept test and the MR Pleiotropy Residual and Outlier method are applied; a *P*-value >.05 in these tests suggests no horizontal pleiotropy. For further analysis, multivariate MR was employed to identify exposure factors that independently had a causal impact on the outcome factor.

#### 2.5.3. Sensitivity analysis

To validate the credibility of the causal effect estimates, a series of sensitivity analyses were conducted. The leave-one-out method was employed to assess the stability of the causal findings, which involved removing one IV at a time and evaluating the effect of each removal on the overall results. If the exclusion of a specific IV substantially altered the outcome, it suggested that the variable was a pivotal element of the MR analysis, or there might have been issues such as genetic bias. Additionally, the MR Pleiotropy Residual and Outlier test, confounding analysis, and heterogeneity tests were carried out to determine whether individual SNPs influenced the assessment of the relationship between exposure and outcome.^[21]^

#### 2.5.4. IV validity and SNP selection

Our MR analysis utilized genome-wide significant SNPs strongly associated with serum zinc levels, satisfying the “relevance” assumption for IVs. The ``exclusion restriction’’ assumption (no pleiotropy) was validated through MR-Egger intercept tests and multivariable MR adjusting for inflammatory biomarkers (CRP, IL-6). SNP-exposure effect directions (β_SNP → Zn) were harmonized with SNP-outcome effects (β_SNP → COPD) using the Mendelian Randomization R package.

## 3. Results

### 3.1. Part of NHANES

#### 3.1.1. Baseline characteristics

Table 1 presents the baseline characteristics of the study population (N = 67,502), categorized into COPD (N = 10,405) and non-COPD groups (N = 57,097). COPD patients were older (mean age: 64.21 vs 55.74 years) and had a higher proportion of females (80.5% vs 52.3%). Non-Hispanic Whites were predominant in the COPD group (72.8% vs 63.6%), with other racial groups showing lower proportions. Zinc exposure was significantly lower in the COPD group (mean: 0.56 vs 0.62, *P* < .001), while smoking prevalence was higher (41.8% vs 39.6%). Differences in marital status, education, and income levels were minimal.

#### 3.1.2. Association between zinc and COPD

Table 2 summarizes the association between zinc exposure (quartiles Q1–Q4) and COPD risk, assessed using weighted logistic regression models. Q1 was the reference group. In the unadjusted Model 1, higher zinc exposure (Q4) significantly reduced COPD risk (OR = 0.824, 95% CI: 0.769–0.882, *P* = .0001). However, after adjusting for demographic, lifestyle, and dietary factors (Models 2 and 3), the association became nonsignificant (OR = 0.980, *P* > .05; File S1, Supplemental Digital Content, https://links.lww.com/MD/P546).

**Table 2:** Results of the quartile-based logistic regression analysis for zinc exposure across 3 models (Model 1, Model 2, Model 3).

Zinc	Model 1		Model 2		Model 3	
	OR 95% CI	*P* value	OR 95% CI	*P* value	OR 95% CI	*P* value
Q1	Ref		Ref		Ref	
Q2	0.918 (0.833, 1.011)	*P* > .05	0.934 (0.865, 1.007)	*P* > .05	0.933 (0.865, 1.007)	*P* > .05
Q3	1.005 (0.912, 1.107)	*P* > .05	1.044 (0.953, 1.145)	*P* > .05	1.044 (0.952, 1.146)	*P* > .05
Q4	0.824 (0.769, 0.882)	*P* = .0001	0.980 (0.918, 1.046)	*P* > .05	0.980 (0.916, 1.047)	*P* > .05
Model 1: adjusted for exposure + Gender + Age + Race + Education.						
Model 2: adjusted for exposure + SEQN + Gender + Age + Race + Education + Marry.						
Model 3: adjusted for exposure + SEQN + Gender + Age + Rce + Education + Marry + income + Smoke.						

This table presents the odds ratios (OR) and 95% confidence intervals (CI) for the association between zinc exposure quartiles (Q2, Q3, Q4) and the outcome, with Q1 serving as the reference group. Results are shown for 3 models: Model 1 (unadjusted), Model 2 (adjusted for demographic and lifestyle factors), and Model 3 (fully adjusted). In Model 1, Q4 demonstrated a significant protective effect (OR = 0.824, 95% CI: 0.769–0.882, *P* = .0001). However, this effect was attenuated in Models 2 and 3 after adjusting for potential confounders.

#### 3.1.3. Analysis of restricted cubic spline regression

**Figure 2.**
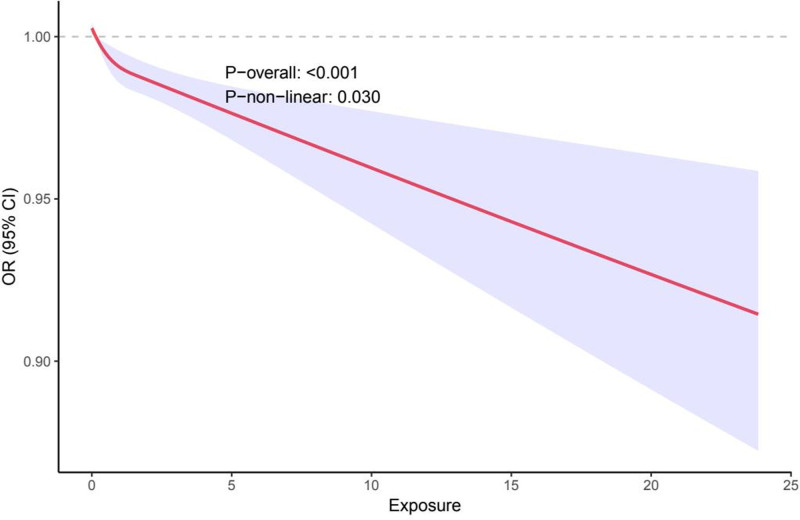
Restricted cubic spline (RCS) analysis depicting the nonlinear relationship between zinc exposure and COPD risk. The solid line represents the odds ratio (OR) for COPD with varying levels of zinc exposure, and the shaded area indicates the 95% confidence interval (CI). A statistically significant overall association (*P*-overall < .001) and a nonlinear trend (*P*-nonlinear = .030) were observed, suggesting a nonlinear dose–response relationship. COPD = chronic obstructive pulmonary disease.

RCS analysis indicated a significant overall association between zinc exposure and COPD risk (*P*-overall < .001), with a nonlinear relationship (*P*-nonlinear = .030; Fig. 2). Odds ratios fluctuated around 1 across exposure levels, suggesting a dose-dependent or threshold effect. The findings imply that both high and low zinc exposure may exert protective effects, though the association is not strictly linear (File S2, Supplemental Digital Content, https://links.lww.com/MD/P546).

#### 3.1.4. Subgroup: association of different zinc with COPD stratified by age

**Figure 3.**
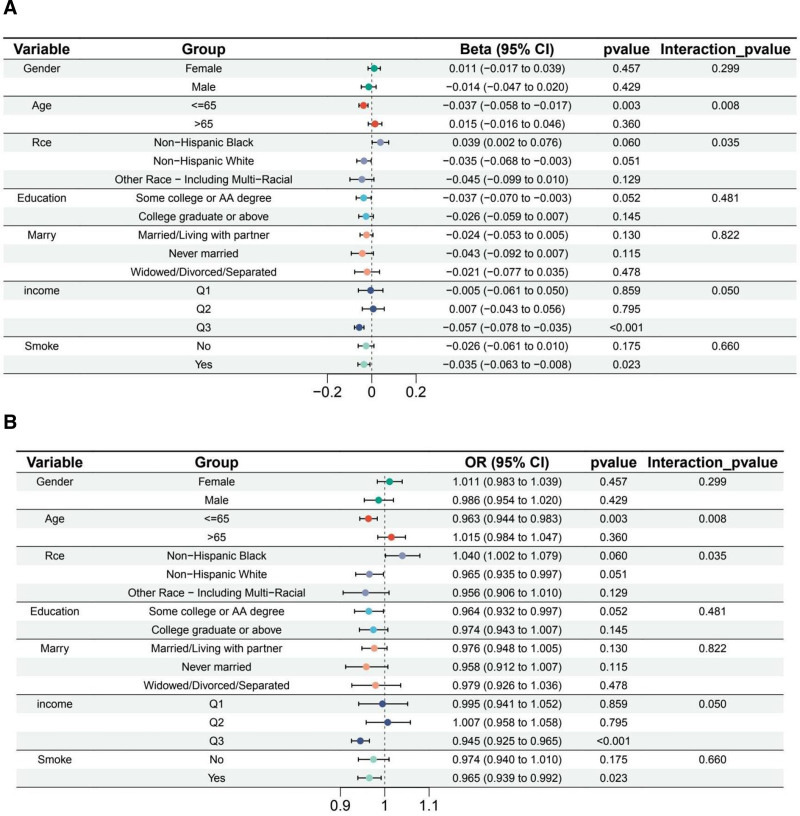
(A) The beta coefficients and 95% confidence intervals for the association between zinc exposure and COPD, stratified by various demographic and socioeconomic factors. Significant protective effects were observed in individuals ≤ 65 years (*P* = .003), Non-Hispanic Whites (*P* = .051), and smokers (*P* = .023). Interaction terms indicated significant differences in zinc’s effects by age (*P* = .008) and race (*P* = .035). (B) The odds ratios (ORs) and 95% confidence intervals for the association between zinc exposure and COPD across stratified groups. Zinc exposure significantly reduced COPD risk in individuals ≤ 65 years (OR = 0.963, *P* = .003), Non-Hispanic Whites (OR = 0.965, *P* = .051), and smokers (OR = 0.965, *P* = .023). Interaction effects were significant for age (*P* = .008) and race (*P* = .035), suggesting subgroup-specific differences in zinc’s protective effects. COPD = chronic obstructive pulmonary disease.

Subgroup analysis revealed that zinc exposure significantly reduced COPD risk in individuals aged ≤ 65 years (beta = ‐0.037, OR = 0.963, *P* = .003), but no significant association was observed in those >65 years. Interaction by age was significant (*P* = .008). Among racial groups, Non-Hispanic Whites showed a marginal protective effect (beta = ‐0.035, OR = 0.965, *P* = .051), while Non-Hispanic Blacks exhibited a borderline positive association (beta = 0.039, OR = 1.040, *P* = .060), with a significant interaction by race (*P* = .035). Smokers benefited significantly from zinc (beta = ‐0.035, OR = 0.965, *P* = .023), but no association was observed among nonsmokers. Income and education had no consistent effects, though individuals in the Q3 income group demonstrated a significant protective effect (beta = ‐0.057, OR = 0.945, *P* < .001; Fig. 3).

### 3.2. Part of Mendelian randomization analysis

#### 3.2.1. Causal relationship between 15 micronutrients and COPD related infections

**Figure 4.**
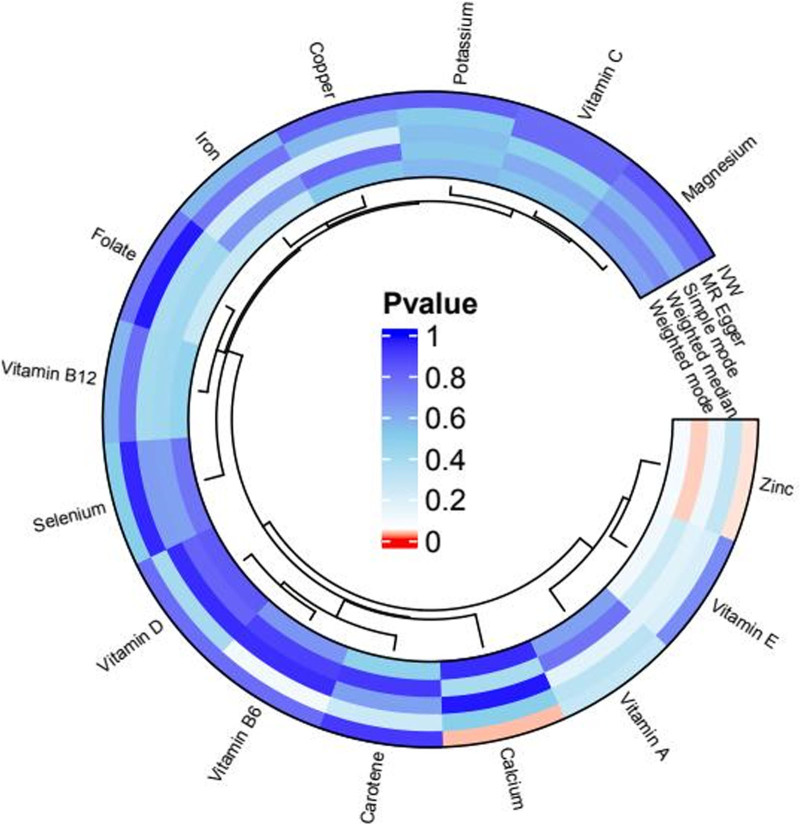
Mendelian randomization analysis of trace elements and vitamin exposure on CRD-related infections. This bar graph summarizes the MR analysis results for various trace elements and vitamins in relation to CRD-related infections. The analysis evaluated carotene, vitamins B6, D, B12, A, E, and C, along with selenium, folate, iron, copper, zinc, calcium, potassium, and magnesium. Each exposure was analyzed using several MR methods, including IVW, MR Egger, simple mode, weighted median, and weighted mode approaches. The plot displays the *P*-values obtained for each nutrient across different MR methods, illustrating the significance of associations. CRD = chronic respiratory disease, IVW = inverse-variance weighted, MR = Mendelian randomization.

A rigorous selection process identified 188 SNPs across 15 micronutrients, including zinc, copper, calcium, and iron, with *F*-statistics ranging from 20.86 to 84.68. These nutrients were treated as exposures, while CRD-related infections served as outcomes (File S3, Supplemental Digital Content, https://links.lww.com/MD/P546). The IVW analysis showed a significant protective effect of zinc (OR = 0.97, 95% CI: 0.958–0.999; *P* = .04) and a positive association with calcium (OR = 1.091, 95% CI: 1.007–1.183; *P* = .03). Other micronutrients, such as selenium and vitamins, did not meet the significance threshold (*P* > .05) (Fig. 4, Files S4 and S5, Supplemental Digital Content, https://links.lww.com/MD/P546).

**Figure 5.**
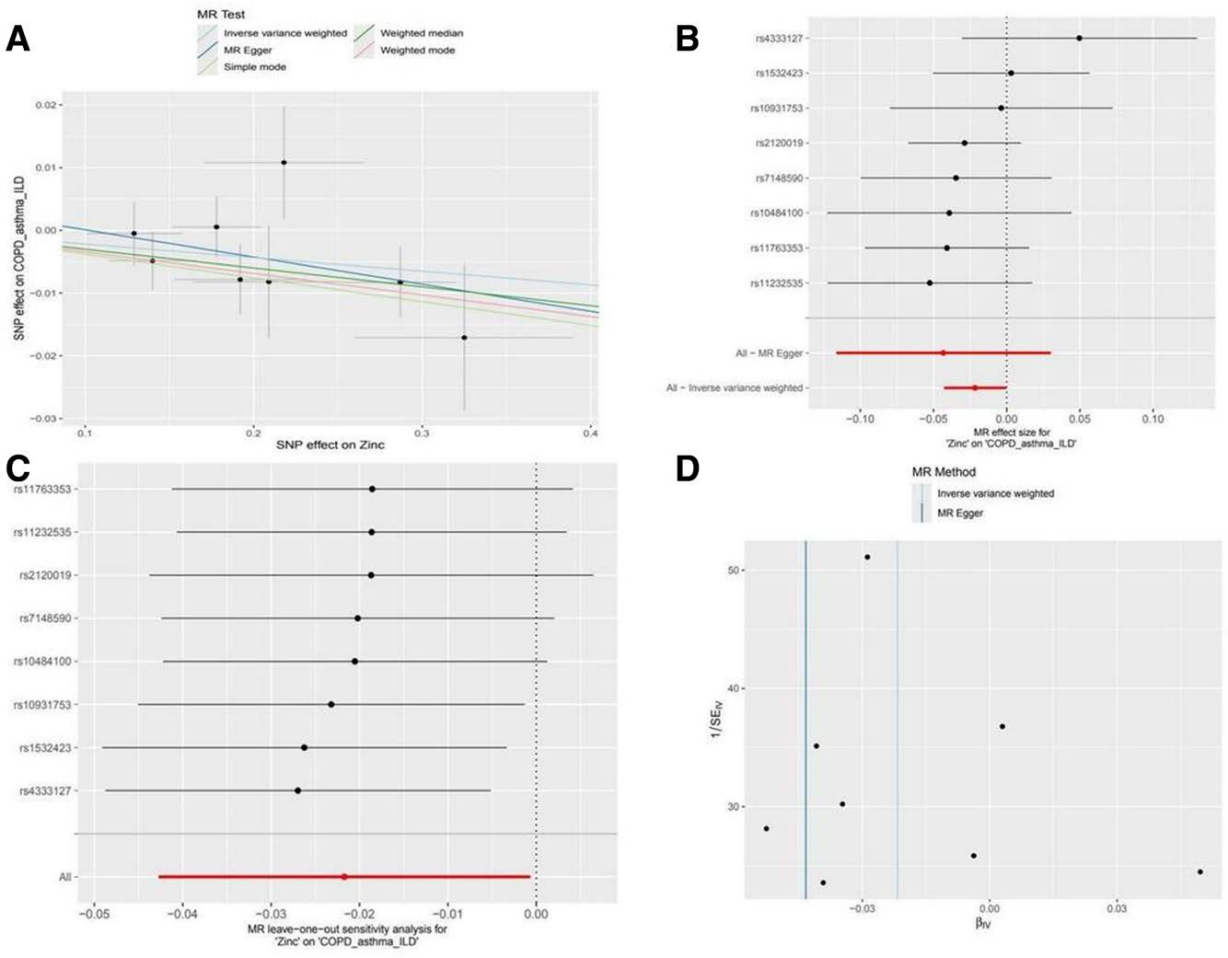
Mendelian randomization analysis of the causal relationship between zinc and CRD-related infections. (A) Scatterplots for the causal association between zinc and CRD-related infections. The slope of a straight line indicates the magnitude of causality. Black dots represent genetic instruments included in the main Mendelian randomization analysis. (B) Forest map visualization of the causal impact of each SNP on CRD-related infections. (C) “Leave-one-out” plots for the causal association between zinc and CRD-related infections. (D) Funnel plot showing heterogeneity of SNP. CRD = chronic respiratory disease, SNP = single nucleotide polymorphisms.

Sensitivity analyses validated zinc’s protective role against COPD-related infections. Tests for confounding and heterogeneity yielded nonsignificant results (*P* > .05; Files S6–S9, Supplemental Digital Content, https://links.lww.com/MD/P546). Forest and funnel plots demonstrated consistent SNP effects, and “leave-one-out” analysis confirmed robust findings. Collectively, the results suggest a causal relationship between zinc and reduced copd-related infection risk (Fig. 5).

#### 3.2.2. Multivariate Mendelian randomization analysis

**Figure 6.**
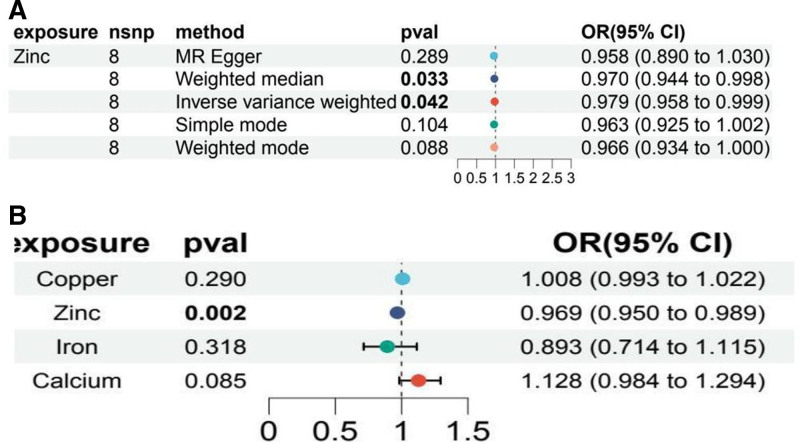
Forest plot. (A) Forest plot of Mendelian randomization analysis of zinc with IVW, weighted median, MR-Egger, simple mode, and weighted mode. (B) Forest plots for inverse variance-weighted Mendelian randomization analysis of copper, iron, calcium and zinc. IVW = inverse variance weighting.

Multivariate MR analysis, adjusted for other trace elements, confirmed zinc as an independent protective factor against COPD-related infections (OR = 0.969, 95% CI: 0.950–0.989; *P* = .002). Calcium (OR = 1.128, 95% CI: 0.983–1.294; *P* = .084), copper (OR = 1.007, 95% CI: 0.994–1.022; *P* = .29), and iron (OR = 0.893, 95% CI: 0.714–1.115; *P* = .318) were not significantly associated. Heterogeneity tests (*P* > .05) indicated no significant multivariate effects (File S11, Supplemental Digital Content, https://links.lww.com/MD/P546). Comparative forest plots highlighted zinc’s distinct role as a protective agent against COPD-related infections (Fig. 6).

### 3.3. MR findings

In the Mendelian randomization analyses, SNPs were utilized as IVs to proxy genetically predicted levels of micronutrients. SNPs associated with higher zinc levels were consistently linked to a reduced risk of COPD-related infections. Specifically, when the genetic variants indicated elevated zinc exposure, the odds of infection decreased (ORs < 1), suggesting a protective effect. Conversely, SNPs linked to lower zinc levels tended to be associated with an increased risk, although not all associations reached statistical significance. This directional consistency supported the robustness of zinc’s causal role. In contrast, SNPs related to other micronutrients, such as calcium and copper, did not show consistent or significant associations, indicating a limited or noncausal role in this context (Files S10 and 11, Supplemental Digital Content, https://links.lww.com/MD/P546).

## 4. Discussion

In this study, we systematically evaluated the association between zinc levels and COPD risk using data from NHANES and MR analyses. Our results demonstrated that higher zinc levels were linked to a lower risk of COPD, with significant nonlinear dose-response patterns observed in restricted cubic spline analyses. Stratified analyses further identified age, race, and smoking status as key modifiers, with the protective effects being most pronounced in individuals aged ≤ 65 years, Non-Hispanic Whites, and smokers. MR analysis supported a causal role for zinc in reducing COPD-related infections, independent of potential confounders, confirming their protective effect across both univariate and multivariate models. Sensitivity analyses reinforced the robustness of these findings, showing no evidence of pleiotropy or heterogeneity.^[22]^

Interestingly, zinc demonstrated distinct maternal transfer dynamics compared to selenium, with only about 60% of maternal zinc being transferred through the placenta by the third trimester, leaving preterm infants particularly vulnerable to deficiency.^[23]^ For instance, a study by Picado et al involving 118 asthma patients found no significant differences in daily micronutrient or antioxidant intake compared to a control group, regardless of asthma severity or plasma/serum zinc levels.^[24]^ In contrast, studies such as Iurina et al^[25]^ have reported a positive correlation between zinc levels and asthma severity. However, our MR-based findings suggest that zinc plays a protective role, as this methodology minimizes confounding and reverse causation, overcoming limitations of observational studies.^[25]^ Consistent with earlier research highlighting zinc’s importance in immune function and respiratory infection reduction,^[26,27]^ our analysis strengthens the evidence for its protective role against COPD and other related infections. Variations in study design, population, and sample size likely explain discrepancies across studies, underscoring the need for large-scale randomized trials.

Deficiencies in zinc and other essential nutrients significantly increase infection risk and weaken immune efficiency. Dietary imbalances often lead to multiple nutrient deficiencies, exacerbating health challenges.^[28]^ Excessive zinc supplementation, however, can disrupt the balance between zinc and copper, 2 closely interacting elements in metabolic pathways.^[29]^ Given the respiratory tract’s constant exposure to pathogens, a robust epithelial barrier is essential for protection.^[30]^ Zinc deficiency undermines this barrier by impairing structural proteins (e.g., β-catenin, E-cadherin) and tight junction proteins (e.g., ZO-1, Claudin-1), increasing permeability and infection susceptibility.^[31,32]^ Additionally, zinc deficiency triggers pro-inflammatory cytokines (interferon-gamma, tumor necrosis factor-alpha) induces apoptosis, further compromising lung barrier function.^[31]^ Zinc plays a pivotal role in regulating immune and inflammatory responses, helping maintain balance and preventing tissue damage.^[33]^ It is essential for the development and activity of immune cells, including monocytes, neutrophils, T cells, B cells, dendritic cells, and natural killer cells, all of which are critical for respiratory defense.^[34]^ Zinc promotes CD8+ T cell proliferation, vital for antiviral responses,^[35,36]^ while its deficiency reduces natural killer cell cytotoxicity, disrupts cytokine production (e.g., IL-1, IL-2, IL-4, interferon-gamma), and impairs the Th1/Th2 balance, collectively weakening immune defense.^[37,38]^

Respiratory epithelial cells form the lungs’ first line of defense against pathogens and environmental insults. Zinc helps maintain this barrier by preventing apoptosis via death receptor pathway inhibition and preserving structural integrity.^[39]^ Inadequate zinc increases apoptosis, degrades junctional proteins, disrupts cell adhesion, and raises epithelial permeability, leaving the lungs vulnerable to damage.^[31]^ Zinc also possesses anti-inflammatory, antioxidant, and antiapoptotic properties, stabilizes organelles, promotes wound healing, and facilitates DNA synthesis, all of which are critical for respiratory health.^[39,40]^ Zinc deficiency compromises these functions and impairs ciliary function in bronchial epithelial cells, reducing cilia length, density, and number, which hinders mucociliary clearance. Conversely, zinc supplementation preserves these functions, enhances immune regulation, and boosts antibacterial activity in monocytes and alveolar macrophages.^[41]^

Despite its strengths, our study has several limitations that should be acknowledged. First, to reduce the risk of population stratification, we stratified data exclusively by European ancestry without adjusting to factors such as age, dietary habits, or micronutrient intake, potentially introducing bias into our findings. Furthermore, due to setting the genome-wide significance threshold at 5 * 10^–8^, we could not identify a sufficient number of SNPs for robust MR analysis. As a result, we expanded the genome-wide significance level to 5 * 10^‐6^; although this adjustment remains within reasonable bounds, it introduces additional limitations. Moreover, exploring gene–environmental interactions, such as dietary habits or exposure to environmental toxins, will provide a more comprehensive understanding of zinc’s protective effects against COPD and related infections.

## 5. Conclusion

This study demonstrated that higher zinc levels were associated with a reduced risk of COPD, with significant nonlinear dose–response patterns. Stratified analyses revealed stronger protective effects in individuals aged ≤ 65 years, Non-Hispanic Whites, and smokers. MR further supported Zinc’s causal role in reducing COPD related infections, independent of confounders. These findings highlight zinc’s importance in respiratory health, emphasizing its potential as a preventative or therapeutic target for COPD.

## Author contributions

**Data curation:** Banghui Liu, Li Peng.

**Formal analysis:** Banghui Liu, Li Peng.

**Investigation:** Banghui Liu, Jinhe Yuan.

**Methodology:** Banghui Liu, Jinhe Yuan, Lu Huan, Li Peng.

**Resources:** Jinhe Yuan.

**Software:** Jinhe Yuan, Lu Huan.

**Supervision:** Lu Huan.

**Validation:** Lu Huan, Li Peng.

**Visualization:** Lu Huan.

**Writing – original draft:** Banghui Liu, Li Peng.

**Writing – review & editing:** Banghui Liu, Li Peng.

## Supplementary Material



## References

[1] Cao B Bray F Ilbawi A Soerjomataram I. Effect on longevity of one-third reduction in premature mortality from non-communicable diseases by 2030: a global analysis of the Sustainable Development Goal health target. Lancet Glob Health. 2018;6:e1288–96. 30420032 10.1016/S2214-109X(18)30411-X

[2] GBD Chronic Respiratory Disease Collaborators. Prevalence and attributable health burden of chronic respiratory diseases, 1990–2017: a systematic analysis for the Global Burden of Disease Study 2017. Lancet Respir Med. 2020;8:585–96. 32526187 10.1016/S2213-2600(20)30105-3 PMC7284317

[3] Li X Cao X Guo M Xie M Liu X. Trends and risk factors of mortality and disability adjusted life years for chronic respiratory diseases from 1990 to 2017: systematic analysis for the Global Burden of Disease Study 2017. BMJ. 2020;368:m234. 32075787 10.1136/bmj.m234 PMC7190065

[4] Christenson SA Smith BM Bafadhel M Putcha N. Chronic obstructive pulmonary disease. Lancet. 2022;399:2227–42. 35533707 10.1016/S0140-6736(22)00470-6

[5] Gombart AF Pierre A Maggini S. A review of micronutrients and the immune system-working in harmony to reduce the risk of infection. Nutrients. 2020;12:236. 31963293 10.3390/nu12010236 PMC7019735

[6] Kinnula VL Crapo JD. Superoxide dismutases in the lung and human lung diseases. Am J Respir Crit Care Med. 2003;167:1600–19. 12796054 10.1164/rccm.200212-1479SO

[7] Cataldo DD Gueders MM Rocks N. Pathogenic role of matrix metalloproteases and their inhibitors in asthma and chronic obstructive pulmonary disease and therapeutic relevance of matrix metalloproteases inhibitors. Cell Mol Biol (Noisy-le-Grand). 2003;49:875–84. 14656045

[8] Truong-Tran AQ Grosser D Ruffin RE Murgia C Zalewski PD. Apoptosis in the normal and inflamed airway epithelium: role of zinc in epithelial protection and procaspase-3 regulation. Biochem Pharmacol. 2003;66:1459–68. 14555222 10.1016/s0006-2952(03)00498-2

[9] Barnett JB Hamer DH Meydani SN. Low zinc status: a new risk factor for pneumonia in the elderly? Nutr Rev. 2010;68:30–7. 20041998 10.1111/j.1753-4887.2009.00253.x PMC2854541

[10] Flatby HM Ravi A Damås JK Solligård E Rogne T. Circulating levels of micronutrients and risk of infections: a Mendelian randomization study. BMC Med. 2023;21:84. 36882828 10.1186/s12916-023-02780-3 PMC9993583

[11] Lassi ZS Kurji J Oliveira CS Moin A Bhutta ZA. Zinc supplementation for the promotion of growth and prevention of infections in infants less than six months of age. Cochrane Database Syst Rev. 2020;4:CD010205. 32266964 10.1002/14651858.CD010205.pub2 PMC7140593

[12] Padhani ZA Moazzam Z Ashraf A. Vitamin C supplementation for prevention and treatment of pneumonia. Cochrane Database Syst Rev. 2020;4:CD013134. 32337708 10.1002/14651858.CD013134.pub2 PMC7192369

[13] Hemani G Zheng J Elsworth B. The MR-Base platform supports systematic causal inference across the human phenome. Elife. 2018;7:1–12. 10.7554/eLife.34408 PMC5976434 29846171

[14] O’Seaghdha CM Wu H Yang Q. Meta-analysis of genome-wide association studies identifies six new Loci for serum calcium concentrations. PLoS Genet. 2013;9:e1003796. 24068962 10.1371/journal.pgen.1003796 PMC3778004

[15] Bell S Rigas AS Magnusson MK. A genome-wide meta-analysis yields 46 new loci associating with biomarkers of iron homeostasis. Commun Biol. 2021;4:156. 33536631 10.1038/s42003-020-01575-z PMC7859200

[16] Auwerx C Sadler MC Woh T Reymond A Kutalik Z Porcu E. Exploiting the mediating role of the metabolome to unravel transcript-to-phenotype associations. Elife. 2023;12:e81097. 36891970 10.7554/eLife.81097 PMC9998083

[17] Li Y Liu H Ye S. The effects of coagulation factors on the risk of endometriosis: a Mendelian randomization study. BMC Med. 2023;21:195. 37226166 10.1186/s12916-023-02881-z PMC10210381

[18] Shen J Zhang H Jiang H. The effect of micronutrient on thyroid cancer risk: a Mendelian randomization study. Front Nutr. 2024;11:1331172. 38496794 10.3389/fnut.2024.1331172 PMC10940541

[19] Voight BF Peloso GM Orho-Melander M. Plasma HDL cholesterol and risk of myocardial infarction: a mendelian randomisation study. Lancet. 2012;380:572–80. 22607825 10.1016/S0140-6736(12)60312-2 PMC3419820

[20] Chalitsios CV Tsilidis KK Tzoulaki I. Psoriasis and COVID-19: a bidirectional Mendelian randomization study. J Am Acad Dermatol. 2023;88:893–5. 36244549 10.1016/j.jaad.2022.10.019 PMC9561435

[21] Li S Chen M Zhang Q Fang M Xiong W Bai L. Ankylosing spondylitis and glaucoma in European population: a Mendelian randomization study. Front Immunol. 2023;14:1120742. 37020551 10.3389/fimmu.2023.1120742 PMC10067563

[22] Burgess S Thompson SG. Interpreting findings from Mendelian randomization using the MR-Egger method. Eur J Epidemiol. 2017;32:377–89. 28527048 10.1007/s10654-017-0255-x PMC5506233

[23] Terrin G Berni Canani R Passariello A. Zinc supplementation reduces morbidity and mortality in very-low-birth-weight preterm neonates: a hospital-based randomized, placebo-controlled trial in an industrialized country. Am J Clin Nutr. 2013;98:1468–74. 24025633 10.3945/ajcn.112.054478

[24] Picado C Deulofeu R Lleonart R. Dietary micronutrients/antioxidants and their relationship with bronchial asthma severity. Allergy. 2001;56:43–9. 10.1034/j.1398-9995.2001.00793.x 11167351

[25] Iurina TM Kupriianova TA Chereĭskaia NK Liamina OI Stotskaia TV. [Macro- and trace elements of blood in elderly patients with bronchial asthma]. Klin Med (Mosk). 2002;80:30–4. 12516337

[26] Malik A Taneja DK Devasenapathy N Rajeshwari K. Zinc supplementation for prevention of acute respiratory infections in infants: a randomized controlled trial. Indian Pediatr. 2014;51:780–4. 25362008 10.1007/s13312-014-0503-z

[27] Shah UH Abu-Shaheen AK Malik MA Alam S Riaz M Al-Tannir MA. The efficacy of zinc supplementation in young children with acute lower respiratory infections: a randomized double-blind controlled trial. Clin Nutr. 2013;32:193–9. 22981241 10.1016/j.clnu.2012.08.018

[28] Plum LM Rink L Haase H. The essential toxin: impact of zinc on human health. Int J Environ Res Public Health. 2010;7:1342–65. 20617034 10.3390/ijerph7041342 PMC2872358

[29] Malavolta M Giacconi R Piacenza F. Plasma copper/zinc ratio: an inflammatory/nutritional biomarker as predictor of all-cause mortality in elderly population. Biogerontology. 2010;11:309–19. 19821050 10.1007/s10522-009-9251-1

[30] Gammoh NZ Rink L. Zinc in infection and inflammation. Nutrients. 2017;9:624. 28629136 10.3390/nu9060624 PMC5490603

[31] Bao S Knoell DL. Zinc modulates cytokine-induced lung epithelial cell barrier permeability. Am J Physiol Lung Cell Mol Physiol. 2006;291:L1132–1141. 16844947 10.1152/ajplung.00207.2006

[32] Roscioli E Jersmann HP Lester S. Zinc deficiency as a codeterminant for airway epithelial barrier dysfunction in an ex vivo model of COPD. Int J Chron Obstruct Pulmon Dis. 2017;12:3503–10. 29255357 10.2147/COPD.S149589 PMC5723110

[33] Duerr CU Fritz JH. Editorial: immunoregulatory mechanisms of interferon. Front Immunol. 2020;11:187. 32117309 10.3389/fimmu.2020.00187 PMC7034390

[34] Prasad AS. Zinc in human health: effect of zinc on immune cells. Mol Med. 2008;14:353–7. 18385818 10.2119/2008-00033.Prasad PMC2277319

[35] Hasan R Rink L Haase H. Zinc signals in neutrophil granulocytes are required for the formation of neutrophil extracellular traps. Innate Immun. 2013;19:253–64. 23008348 10.1177/1753425912458815

[36] Hasan R Rink L Haase H. Chelation of free Zn^2+^ impairs chemotaxis, phagocytosis, oxidative burst, degranulation, and cytokine production by neutrophil granulocytes. Biol Trace Elem Res. 2016;171:79–88. 26400651 10.1007/s12011-015-0515-0

[37] Hojyo S Fukada T. Roles of zinc signaling in the immune system. J Immunol Res. 2016;2016:6762343. 27872866 10.1155/2016/6762343 PMC5107842

[38] Kido T Ishiwata K Suka M Yanagisawa H. Inflammatory response under zinc deficiency is exacerbated by dysfunction of the T helper type 2 lymphocyte-M2 macrophage pathway. Immunology. 2019;156:356–72. 30552817 10.1111/imm.13033 PMC6418430

[39] Truong-Tran AQ Carter J Ruffin R Zalewski PD. New insights into the role of zinc in the respiratory epithelium. Immunol Cell Biol. 2001;79:170–7. 11264713 10.1046/j.1440-1711.2001.00986.x

[40] Stafford SL Bokil NJ Achard MES. Metal ions in macrophage antimicrobial pathways: emerging roles for zinc and copper. Biosci Rep. 2013;33:e00049. 23738776 10.1042/BSR20130014 PMC3712485

[41] Liu MJ Bao S Gálvez-Peralta M. ZIP8 regulates host defense through zinc-mediated inhibition of NF-κB. Cell Rep. 2013;3:386–400. 23403290 10.1016/j.celrep.2013.01.009 PMC3615478

